# The effects of misclassification in routine healthcare databases on the accuracy of prognostic prediction models: a case study of the CHA2DS2-VASc score in atrial fibrillation

**DOI:** 10.1186/s41512-017-0018-x

**Published:** 2017-11-16

**Authors:** S. van Doorn, T. B. Brakenhoff, K. G. M. Moons, F. H. Rutten, A. W. Hoes, R. H. H. Groenwold, G. J. Geersing

**Affiliations:** Julius Center for Health Sciences and Primary care, University Medical Center Utrecht, PO box 85500, 3508 AB Utrecht, The Netherlands

**Keywords:** Routine care data, Validation, Prediction model, Atrial fibrillation, CHA2DS2-VASc, Misclassification

## Abstract

**Background:**

Research on prognostic prediction models frequently uses data from routine healthcare. However, potential misclassification of predictors when using such data may strongly affect the studied associations. There is no doubt that such misclassification could lead to the derivation of suboptimal prediction models. The extent to which misclassification affects the validation of existing prediction models is currently unclear.

We aimed to quantify the amount of misclassification in routine care data and its effect on the validation of the existing risk prediction model. As an illustrative example, we validated the CHA2DS2-VASc prediction rule for predicting mortality in patients with atrial fibrillation (AF).

**Methods:**

In a prospective cohort in general practice in the Netherlands, we used computerized retrieved data from the electronic medical records of patients known with AF as index predictors. Additionally, manually collected data after scrutinizing all complete medical files were used as reference predictors. Comparing the index with the reference predictors, we assessed misclassification in individual predictors by calculating Cohen’s kappas and other diagnostic test accuracy measures. Predictive performance was quantified by the c-statistic and by determining calibration of multivariable models.

**Results:**

In total, 2363 AF patients were included. After a median follow-up of 2.7 (IQR 2.3–3.0) years, 368 patients died (incidence rate 6.2 deaths per 100 person-years). Misclassification in individual predictors ranged from substantial (Cohen’s kappa 0.56 for prior history of heart failure) to minor (kappa 0.90 for a history of type 2 diabetes). The overall model performance was not affected when using either index or reference predictors, with a c-statistic of 0.684 and 0.681, respectively, and similar calibration.

**Conclusion:**

In a case study validating the CHA2DS2-VASc prediction model, we found substantial predictor misclassification in routine healthcare data with only limited effect on overall model performance. Our study should be repeated for other often applied prediction models to further evaluate the usefulness of routinely available healthcare data for validating prognostic models in the presence of predictor misclassification.

**Electronic supplementary material:**

The online version of this article (10.1186/s41512-017-0018-x) contains supplementary material, which is available to authorized users.

## Background

Prognostic prediction models aim to estimate the probability that a certain outcome may develop in the future and, in many medical fields, they are essential in assisting clinical decision making. Studies on prediction models include development, validation, updating, and implementation and frequently rely on large datasets from routine healthcare [1]. Derived from, for instance, electronic health records or administrative databases, these data offer great potential for clinical research. After a prediction model is developed and its potential usefulness is recognized, it is typically validated, possibly using routine healthcare data, in different healthcare settings and various countries to justify its application.

Yet, while the validity of routine healthcare data [2] and implications of potential misclassification on studied associations [3–6] are well-addressed *in general*, misclassification in predictors in the context of prognostic research *specifically* has received little attention. Even though the RECORD statement [2] suggests to assess the accuracy of categorical routine healthcare variables by comparing them to a reference standard using diagnostic test accuracy measures (i.e., sensitivity, specificity, positive and negative predictive values) or kappa coefficients, it is still unknown whether this approach sufficiently captures the potential bias and/or imprecise inferences that may arise when validating existing prediction models.

Using the well-known CHA2DS2-VASc model as a case study, we aimed to further explore the influence of predictor misclassification on the validation of a prediction model when using routine healthcare or registry data.

First, we quantified the amount of misclassification present in routine care registry data of a representative sample of patients with atrial fibrillation in general practice. Second, we assessed the influence of predictor misclassification on the accuracy of the CHA2DS2-VASc model to predict mortality when validated on such data.

## Methods

### Clinical setting and the CHA2DS2-VASc prediction rule

Atrial fibrillation is the most common cardiac arrhythmia, with a prevalence of 1–2% in the general population [7]. It is a major risk factor for ischemic stroke; hence, the prediction (and subsequent reduction) of stroke risk is a mainstay in the treatment of atrial fibrillation [8]. Practice guidelines [9–11] recommend the use of a clinical prediction rule, of which the CHA2DS2-VASc rule is now most commonly recommended and used. This rule was developed in 2010 by Lip et al. [12], as an update to the earlier CHADS2 score [13], and originally intended to predict either an ischaemic stroke, peripheral embolism, or pulmonary embolism by assigning AF patients points for congestive heart failure (1 point), hypertension (1 point), age above 75 years (2 points), diabetes (1 point) and prior stroke (2 points), age above 65 (1 point), vascular disease (1 point), and female sex (1 point). The total score subsequently results in an expected annual stroke risk (see Tables 1 and 2).

**Table 1:** The original CHA2DS2-VASc score [12]

Predictor	Score
Congestive heart failure/LV dysfunction	1
Hypertension	1
Age ≥ 75 years	2
Diabetes mellitus	1
Stroke/TIA/TE	2
Vascular disease (prior myocardial infarction, peripheral artery disease, or aortic plaque)	1
Age 65–74 years	1
Sex category (i.e., female sex)	1

*TE* thromboembolism

**Table 2:** The annual risks of thromboembolism (ischemic stroke, peripheral embolism, or pulmonary embolism) for CHA2DS2-VASc, adjusted for aspirin use [12]

CHA2DS2-VASc score	Risk (events/persons)
0	0 (0/103)
1	0.7 (1/162)
2	1.9 (3/184)
3	4.7 (8/203)
4	2.3 (4/208)
5	3.9 (3/95)
6	4.5 (2/57)
7	10.1 (2/25)
8	14.2 (1/9)
9	100 (1/1)

The original study deriving the CHA2DS2-VASc consisting of 1084 AF patients with a follow-up of 1 year, considering ischemic stroke, peripheral embolism, or pulmonary embolism as outcomes for thromboembolism

### Index predictors: routine care ICPC codes

We used data from the CAFe study, a large prospective cohort study of patients with atrial fibrillation in general practice in the Netherlands aimed to validate the accuracy of the CHA2DS2-VASc prediction model and to quantify the effect of an automated treatment decision support tool (trial registration number NTR3741) in a cluster randomized trial. From February 2013 until September 2014, 38 general practices were enrolled. All patients with electrocardiographically confirmed atrial fibrillation were included in the CAFe cohort. Follow-up lasted a minimum of 2 years. Every 3 months, the electronic patient file of these AF patients was captured into a designated research database, containing diagnosis codes, and free text records and test results. In the Netherlands, general practitioners (GPs) are encouraged to record “diagnosis codes” according to the International Classification of Primary Care (ICPC) [14] during routine care consultations. In the general practices, personal details are registered through linkage to administrative data from the municipal authorities, of which age and sex are captured into the research database. For the remaining predictor values in CHA2DS2-VASc, the corresponding ICPC codes were automatically retrieved and considered as the index predictors. For an overview of the ICPC codes used, see Table 3.

**Table 3:** Automatically extracted ICPC codes for the index predictors in the CHA2DS2-VASc model and the definition of the reference predictors used for manually scrutinizing the electronic patient file

Predictor	ICPC code(s) for index predictors	Definition for reference predictors
Congestive heart failure	K77 heart failure	Signs and symptoms suggestive of heart failure, with structural or functional abnormalities on echocardiography, either with preserved or reduced ejection fraction
Hypertension	K86 hypertension without organ damage K87 hypertension with organ damage/secondary hypertension	Repeated systolic blood pressure measurement of 140 mmHg or higher
Age	Age in years	Age in years
Diabetes	T90 type 1 and type 2 diabetes	Repeated fasting blood glucose measurement of ≥ 7.0 mmol/L (126 mg/dL) or a non-fasting glucose measurement of ≥ 11.1 mmol/L (200 mg/dL)
Stroke/TIA	K89 TIA K90 cerebrovascular accident (stroke)	Focal neurological deficit of sudden onset lasting > 24 or < 24 h, respectively
Vascular disease	K74 angina pectoris K75 acute myocardial infarction K76 other chronic ischemic heart disease K91 atherosclerosis K92 other peripheral arterial disease K03 other pain suspected to originate from the cardiovascular tract	• Coronary heart disease: prior myocardial infarction (both ST-elevated myocardial infarction or non-ST-elevated myocardial infarction), angina pectoris or prior percutaneous coronary intervention (PCI) or coronary artery bypass graft surgery (CABG) • Peripheral artery disease: symptoms of intermittent claudication with ankle-branchial index ≤ 0.9 or prior surgery or percutaneous intervention on the abdominal or thoracic aorta or lower extremity vessels Previous thrombo-embolism
Sex category	Female sex	Female sex

### Reference predictors: manually verified predictors

Except for the predictors “Age” and “Sex category,” which were obtained from the municipal authorities, the correctness of the routinely recorded ICPC codes corresponding to the remaining CHA2DS2-VASc predictors was manually checked using all available information from the electronic patient file including diagnostic test results, out-of-hours office reports, and specialists’ letters. As such, each patient file was thoroughly scrutinized and the value of each ICPC code corresponding to the predictors in the CHA2DS2-VASc was recorded. These values were collectively used as the reference predictors of which the definitions are shown in Table 3.

For each patient, two values for the CHA2DS2-VASc predictors were included in the dataset: one based on the ICPC codes recordings (index) and one based on the manual check of these ICPC codes by scrutinizing the complete patient file (reference).

### Outcome

Our aim was to study potential misclassification in the prediction variables, not in the outcome. The CHA2DS2-VASc was originally developed to predict either ischemic stroke, peripheral embolism, or pulmonary embolism. In our methodological study, however, we used all-cause mortality as an illustrational outcome for two reasons. First, stroke may be difficult to diagnose, especially stroke as the cause of (unexpected) death. The outcome all-cause mortality can be objectively determined. Second, such mortality data may often be captured by the municipal authorities, as was the case for the general practices in our study, further avoiding misclassification in the outcome. We manually checked vital status using the electronic patient file. Follow-up was a minimum of 2 years.

### Data analyses

The following analyses were performed to assess misclassification in the predictors based on routinely recorded ICPC codes (index) and determine the consequences of such misclassification on the prediction of all-cause mortality:

1. We compared the index predictor values with the reference predictor values using Cohen’s kappa [15] and calculated sensitivity, specificity, and positive and negative predictive values of the dichotomous index predictors with respect to the reference predictors.
2. For each patient, we calculated the CHA2DS2-VASc score using either the index predictors or the reference predictors. We tabulated the two distributions of these CHA2DS2-VASc scores and the discordance. Next, for each score on CHA2DS2-VASc based on index predictors and reference predictors, we calculated the mortality incidence rate (IR) per 100 person-years.
3. To assess the influence of misclassification on discrimination, we calculated the c-statistic using censored data for the CHA2DS2-VASc model as a continuous point-based score based on the index predictors and on the reference predictors.
4. For assessing the influence of misclassification on calibration, data on the baseline hazard and hazard ratios for the CHA2DS2-VASc model predicting mortality are missing. To obtain these, we first fitted a multivariable Cox proportional hazards CHA2DS2-VASc model using the individual reference predictors. We assessed calibration by creating a calibration plot and calculating the calibration slope. Using the same baseline hazard and hazard ratios, we then assessed calibration using the index predictors; the difference in calibration then occurring can only be caused by misclassification.

All analyses were performed in *R* [16] version 3.32 with the packages *survival* 2.40–1 and *rms* 5.1–0.

## Results

A total of 2363 patients with atrial fibrillation were included in the cohort. The median age was 77 (IQR 68–84) years, and 52.3% were male. During a follow-up of 5901 person-years (median 2.7 years, IQR 2.3–3.0), in total, 368 patients died (crude incidence rate 6.2/100 person-years), mostly from non-cardiac causes (74%).

### Misclassification in individual predictor values

There was substantial variation in the amount of misclassification between the index predictors (see Table 4). For instance, the prevalence of (a history of) heart failure according to the ICPC codes was 28.1%, whereas by manually checking all available information in the electronic patient file, the prevalence was 18.3% (Cohen’s kappa 56.1). The prevalence of other index and reference predictors were more comparable, e.g., for hypertension, 60.8 and 59.9% (kappa 70.9), respectively, and for diabetes, 24.3 and 22.5% (kappa 89.7), respectively. For cross tables with the presence and absence of each predictor individually, see Additional file 1: Table S1. Sensitivity (i.e., the proportion of patients with heart failure according to the reference predictor that correctly had the diagnosis according to the index predictor) was lowest for heart failure (55%) and highest for diabetes (89%). Specificity (i.e., the proportion of patients without heart failure according to the reference predictor that correctly were diagnosed as such using the index predictor) ranged from 83% (hypertension) to 99% (diabetes). A similar pattern was observed for the predicted probabilities. Diabetes showed the highest PPV (i.e., the probability of having diabetes according to the reference predictor if diagnosed with diabetes according to the index predictor) and NPV (i.e., the probability of not having diabetes according to the reference predictor if the index predictor was absent) of 98.8 and 96.4%, respectively. Hypertension again showed the lowest values (83.3 and 81.6%, respectively).

**Table 4:** Prevalence of individual ICPC codes (index predictors) and manually verified diagnoses (reference predictors) and measures of misclassification

	ICPC codes (index predictors)	Manually verified diagnoses (reference predictors)	Kappa	Sensitivity	Specificity	PPV	NPV
Congestive heart failure/LV dysfunction	28.1	18.3	56.1	54.5	95.7	83.3	84.3
Hypertension	60.8	59.9	70.9	87.8	83.3	89.1	81.6
Diabetes	24.3	22.5	89.7	88.6	98.8	95.8	96.4
Stroke/TIA/TE	18.7	16.4	75.5	74.8	97.1	85.5	94.4
Vascular disease	34.6	26	60.4	63.1	93.7	84.2	82.7

*ICPC* International Classification of Primary Care, *PPV* positive predictive value, *NPV* negative predictive value

### CHA2DS2-VASc scores and observed mortality

**Fig. 1:**
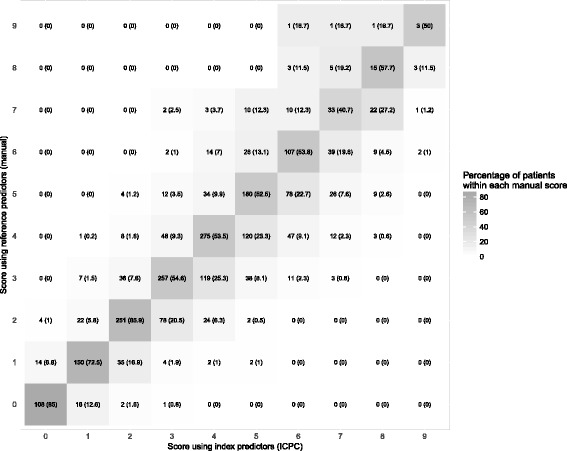
The concordance of CHA2DS2-VASc scores as calculated using the index predictors (*x*-axis) and as calculated using the reference predictors (*y*-axis). Numbers are counts (percentages)

With respect to the reference predictors, the index predictors assigned patients the correct CHA2DS2-VASc scores in between 40.7% (for score 7) and 85.0% (for score 0); see Fig. 1. The median CHA2DS2-VASc score using index data was 4.0 (IQR 2–5); for the reference data, this was 3.0 (IQR 2–5).

Table 5 shows the number of patients, the number of events, the total number of person-years, and the observed IR of all-cause mortality for each CHA2DS2-VASc score calculated with index and reference predictors. Although small numbers for the lowest and highest score limit definite conclusions, we observed a relative ~ 10% difference between both sets of predictors. For instance, for patients with a score 4 according to the index predictors, the IR was 5.6 per 100 person-years, while this was 6.5 per 100 person-years for the same score according to the reference predictors.

**Table 5:** Incidence rate of all-cause mortality for each CHA2DS2-VASc score as calculated with ICPC codes (index predictors) or manually verified diagnoses (reference predictors)

	ICPC codes (index predictors)				Manually verified diagnoses (reference predictors)			
Score	No. of patients (%)	No. of events	py	IR	No. of patients (%)	No. of events	py	IR
0	124 (5.3)	2	338	0.6	125 (5.3)	2	346	0.6
1	194 (8.2)	2	541	0.4	203 (8.6)	4	567	0.7
2	307 (13.0)	29	892	3.3	344 (14.6)	37	994	3.7
3	356 (15.1)	48	1041	4.6	417 (17.7)	54	1208	4.5
4	404 (17.2)	67	1186	5.6	431 (18.3)	83	1274	6.5
5	292 (12.4)	86	887	9.7	254 (10.8)	89	795	11.2
6	187 (7.9)	70	590	11.9	139 (5.9)	60	441	13.6
7	82 (3.5)	37	262	14.1	54 (2.3)	27	187	14.4
8	33 (1.4)	26	127	20.5	16 (0.7)	10	60	16.7
9	8 (0.3)	1	23	4.3	4 (0.2)	2	14	14.3

*py* person-years/100, *IR* incidence rate no. as of events/100 person-years

### Discrimination

The c-statistics were 0.685 (95% CI 0.655–0.715) for the CHA2DS2-VASc model as a continuous score based on the index predictors and 0.682 (95% CI 0.653–0.712) based on the reference predictors, respectively.

### Calibration

To obtain a baseline hazard and hazard ratios of CHA2DS2-VASc predicting mortality, we first fitted a multivariable Cox proportional hazards with the individual reference predictors. Details on this model are specified in Additional file 1: Table S2. The calibration slope of this model was 1.00 (95% CI 0.85–1.15).

**Fig. 2:**
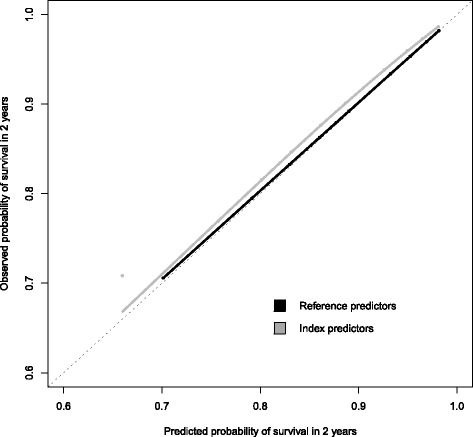
Calibration plot showing deciles of observed and predicted probabilities of survival from the CHA2DS2-VASc model developed using the reference predictors and validated using the baseline hazard and coefficients for validation with the index predictors as input values

The baseline hazard and hazard ratios were then used to assess calibration using the index predictors. There was a slight underestimation of the probability of survival across all risk deciles when using routine healthcare data, though differences in calibration were minimal with equal calibration slope of 1.00 (95% CI 0.86–1.15). See Fig. 2.

## Discussion

We illustrated the impact of potential misclassification in routine healthcare data when such data was used as predictors in a prognostic prediction model. In our validation of the CHA2DS2-VASc rule in patients with atrial fibrillation, we found substantial misclassification in the predictor values from routinely collected general practice diagnosis codes, but this did not affect the accuracy of the model to predict mortality.

In recent years, the availability of data routinely collected during healthcare delivery has grown substantially [17], whereas in the past epidemiologic research often was dependent on dedicated prospective cohorts [18]. With the availability of faster computers and software programs, everyday healthcare data, possibly linked to other data sources, has a great potential for large-scale observational clinical studies. Indeed, in the field of atrial fibrillation, for instance, studies evaluating populations with over 100,000 AF patients are becoming the new standard, rather than an exception [19–21]. Importantly though, these studies mostly rely on diagnosis disease codes (e.g., ICD-10 codes, READ codes, or ICPC coding) as generated during daily clinical practice. Following studies that investigated the completeness of morbidity coding [22] or the methods and reporting of validity assessment [23], the quality of these data has been questioned. While these studies certainly contribute to knowledge on the validity of routine healthcare *data itself*, it does not provide full insight in the validity of applying such data in prediction models. This is important, because the number of prediction models used in everyday practice is rapidly increasing [24–26].

To the best of our knowledge, our study is the first to quantify the influence of predictor misclassification in these data on the results of a study validating a clinical prediction model.

For full appreciation of our findings, several remarks should be made. First, several processes leading to misclassification in data from routine healthcare can be hypothesized. At the most basic level, simple coding mistakes such as typing errors or choosing the wrong diagnosis code may lead to the inadvertent presence or absence of a diagnosis code. Furthermore, if an initially suspected disease (e.g., heart failure or coronary heart disease) is not confirmed after future diagnostic testing, the diagnosis code needs active removal from the electronic patient file or it will lead to “false positives.” Practitioners conversely may also omit diagnosis codes for certain diseases frequently occurring and managed concomitantly. For instance, recording “hypertension” and “coronary heart disease” (both included in the CHA2DS2-VASc model) together as “cardiovascular disease” may cause “false negatives” in the index predictors.

Second, a further cause for misclassification may be suboptimal diagnostic criteria for a certain disease. We found substantial variation in the validity of data from routine healthcare where, for instance, “a history of heart failure” showed notable misclassification. It can be difficult to diagnose heart failure, especially in absence of echocardiography as is often the case in general practice. Indeed it has been shown that heart failure is often over-diagnosed in general practice, similarly as in our study [27]. Diabetes, on the contrary, is predominantly diagnosed in general practice based on well-defined diagnostic criteria and showed very limited misclassification. When using routine care data in epidemiological research, potential difficulties in diagnosis of diseases and thus variation between data sources in the variables under study (e.g., electronic patient records or administrative databases) should be considered [28].

Third, the CHA2DS2-VASc score is a simplistic decision rule, with limited integer weighting of predictors (1 of 2 points). Although we did find pronounced differences in the score as calculated with index or reference predictors, using such simple weighting could also have “canceled out” some of the misclassification. Future studies should investigate the effects of misclassification in predictors on the predictive performance of other prediction models.

Fourth, as a result of misclassification in predictors, the total CHA2DS2-VASc score for a given patient differed substantially between data sources. This may have large implications if a cut-point is applied as is the case with the CHA2DS2-VASc score [9]. Well-defined specific treatment recommendations apply for those with a score of 0, 1, or ≥ 2, and miscalculation by only one point will impact the proportion of patients eligible for anticoagulant treatment. As an illustration of the patients in whom such treatment was indicated (CHA2DS2-VASc score ≥ 2) based on index predictors, nearly 20% had a score of 0–1 based on reference predictors and thus no strict indication for treatment. Likewise, validation studies of prediction rules commonly report the observed risk per score, and in our study, there was a ~ 10% relative difference for many CHA2DS2-VASc scores, though the numbers of events often were small.

Lastly, while misclassification in individual predictors was substantial, the discrimination and calibration of full models containing all predictors of CHA2DS2-VASc was comparable between routinely collected index data and the reference data. The misclassification in the former, thus, seem to “average out” in multivariable analyses. Our results suggest that while a data source shows low performance on the “traditional” measures of accuracy (kappa, sensitivity/specificity, and predictive values), one may still observe valid estimates when validating a multivariable prediction model.

### Strengths and limitations

Strengths of our study include the opportunity to assess misclassification in predictors from routine healthcare from the well-known CHA2DS2-VASc model. This model is recommended by multiple practice guidelines and frequently validated using data from routine healthcare. We verified the disease status, predictor values, and outcomes in a large sample of over 2000 health records. Manually scrutinizing electronic patients files is a resource-intensive process, and we believe this amount approaches what may be considered the maximum realistically feasible. Furthermore, we could collect clinical data from general practice but also could include specialists’ letters with diagnoses and test results from secondary care. Consequently, we were able to study an often used clinical prediction rule without any missing data.

A limitation of our study is that, irrespective of clear definitions for manually checking the predictors, some information (e.g., description of signs and symptoms in free text fields) leave room for different interpretation. The final judgment was made by the researcher, based essentially on the same data that was used by the GP to record the initial ICPC diagnosis code. We did not subject patients to any new clinical assessment. As such, some misclassification might also have occurred in our reference data. Furthermore, we only evaluated a single prediction model. How our results apply to other prognostic prediction models should be the focus of future research. In addition, our study used all-cause mortality as the outcome, while the CHA2DS2-VASc rule was specifically designed to predict stroke risk. While this avoided misclassification in the outcome, the influence of misclassification on the performance of its intended purpose requires further research. Last, it should be stressed that we only focus on the validation of a prediction model. For prediction model development using Cox analysis, methods on how to correct for misclassification in predictors have been previously addressed [29].

### Future considerations

Our results provide evidence that misclassification in routine healthcare data can be substantial and that several aspects (e.g., the risk of the outcome with a certain score) of the validation of a clinical prediction rule may be influenced, while other aspects (such as discrimination and calibration) may not. Future studies should focus on the influence of misclassification on the predictive performance of more complex models, or the influence of different predictor misclassification patterns, e.g., using a simulation study. In addition, when data on true predictor status is available, this can be used to correct for misclassification in routine healthcare data [5]. Insight is needed in the amount of reference data necessary to ensure reliable prediction model performance. This can advise researchers on the efforts required to obtain any reference data (e.g., the proportion of patients’ files that needs manual checking). Ultimately, future research on these topics can further inform applied researchers on when routine healthcare data can reliably be used to evaluate prediction models.

## Conclusion

In this case study of CHA2DS2-VASc, we observed that even in the presence of substantial predictor misclassification in routine healthcare data, the overall performance of a prediction model was not negatively affected.

## Additional file

Additional file 1: Table S1. Cross tables with the presence and absence of each index and reference predictor; **Table S2.** Details of the Cox proportional hazards model predicting mortality using the index predictors. (DOCX 66 kb)

## Abbreviations

GP: General practitioner
ICPC: International Classification of Primary Care
IQR: Interquartile range
