# Supplementary material for: The effects of misclassification in routine healthcare databases on the accuracy of prognostic prediction models: a case study of the CHA2DS2-VASc score in atrial fibrillation
Source: Diagn Progn Res. 2017 Nov 16;1:18. doi: 10.1186/s41512-017-0018-x (PMC6460749; doi:10.1186/s41512-017-0018-x)
Supplement: Supplementary file 1 — Cross tables with the presence and absence of each index and reference predictor; Table S2. Details of the Cox proportional hazards model predicting mortality using the index predictors. (DOCX 66 kb) [file 41512_2017_18_MOESM1_ESM.docx]

**Table S1. Cross tables with the presence and absence of each index and reference predictor**

|  |  |  | **Index predictor** | |
| --- | --- | --- | --- | --- |
|  |  |  | **0** | **1** |
| **Reference predictor** | **Heart failure** | **0** | 1622 (68.9) | 301 (12.8) |
|  |  | **1** | 72 (3.1) | 360 (15.3) |
|  |  |  |  |  |
|  | **Hypertension** | **0** | 770 (32.7) | 174 (7.4) |
|  |  | **1** | 154 (6.5) | 1257 (53.4) |
|  |  |  |  |  |
|  | **Diabetes** | **0** | 1761 (74.8) | 65 (2.8) |
|  |  | **1** | 22 (0.9) | 507 (21.5) |
|  |  |  |  |  |
|  | **Stroke/TIA/TE** | **0** | 1858 (78.9) | 111 (4.7) |
|  |  | **1** | 56 (2.4) | 330 (14) |
|  |  |  |  |  |
|  | **Vascular disease** | **0** | 1442 (61.2) | 301 (12.8) |
|  |  | **1** | 97 (4.1) | 515 (21.9) |

0 = predictor absent

1 = predictor present

numbers are counts (percentages)

**Table S2. Details of the Cox proportional hazards model predicting mortality using the index predictors.**

| **Predictor** | **Coefficient** | **s.e.** |
| --- | --- | --- |
| **Congestive heart failure/LV dysfunction** | 0.53 | 0.11 |
| **Hypertension** | -0.17 | 0.11 |
| **Age ≥75 years** | 1.92 | 0.3 |
| **Diabetes mellitus** | 0.27 | 0.12 |
| **Stroke/TIA/TE** | 0.49 | 0.12 |
| **Vascular disease** | 0.33 | 0.11 |
| **Age 65-74 years** | 0.91 | 0.32 |
| **Sex category (i.e. female sex)** | 0.1 | 0.11 |

s.e = standard error
